# Association of CYBA gene (-930 A/G and 242 C/T) polymorphisms with oxidative stress in breast cancer: a case-control study

**DOI:** 10.7717/peerj.5509

**Published:** 2018-10-04

**Authors:** Mohini A. Tupurani, Chiranjeevi Padala, Kaushik Puranam, Rajesh K. Galimudi, Keerthi Kupsal, Nivas Shyamala, Srilatha Gantala, Ramanjaneyulu Kummari, Sanjeeva K. Chinta, Surekha R. Hanumanth

**Affiliations:** 1 Department of Genetics & Biotechnology, Osmania University, Hyderabad, Telangana, India; 2 Department of Radiation Oncology, MNJ Institute of Oncology Regional Cancer Center, Hyderabad, Telangana, India

**Keywords:** Oxidative stress, CYBA, LD, Haplotype, Insilco analysis, Polymorphism, MDR

## Abstract

**Background:**

Oxidative stress (OS) is a key characteristic feature in cancer initiation and progression. Among multiple cancers, NADPH oxidase (NOX) dependent free radical production is implicated in oxidative stress. P22phox, a subunit of NADPH oxidase encoded by the CYBA gene has functional polymorphisms associated with various complex diseases. The present study was aimed to examine the importance and association of the functional polymorphisms of CYBA gene (-930 A/G and 242 C/T) with the oxidative stress in breast cancer (BC) development and progression.

**Materials and Methods:**

We have performed a case-control study on 300 breast cancer patients and 300 healthy individuals as controls to examine the role of CYBA gene -930 A/G and 242 C/T single nucleotide polymorphisms (SNPs) using As-PCR and PCR-RFLP assays and its association with OS as measured by plasma MDA levels. Linkage disequilibrium (LD) plots were generated using Haploviewtool and Multifactor dimensionality reduction (MDR) analysis was applied to assess high-order interactions between the SNPs. The Insilco analysis has been performed to predict the effect of SNPs on the gene regulation using online tools.

**Results:**

We have found that genotype frequencies of CYBA gene -930 A/G and 242C/T polymorphism were significantly different between controls and BC patients (*p* < 0.05). The haplotype combination -930G/242C and -930G/242T were associated with 1.44 & 1.56 folds increased risk for breast cancer respectively. Further, the MDA levels were higher in the patients carrying -930G/242C and -930G/242T haplotype (*p* < 0.001). Our results have been substantiated by Insilco analysis.

**Conclusion:**

Results of the present study suggest that GG genotype of -930 A/G polymorphism, -930G/242C and -930G/242T haplotypes of CYBA gene polymorphisms have shown association with higher MDA levels in breast cancer patients, signify that elevated oxidative stress might aid in increased risk for breast cancer initiation and progression.

## Introduction

Breast cancer (BC) is one of the most frequent malignant tumors, and its morbidity and mortality rates have been increasing in developing countries such as, India (Gupta, Shridhar & Dhillon, 2015). The breast cancer etiology is complex, involves dynamic interactions of genetic and environmental factors (Abdulkareem, 2013).

Oxidative stress (OS) is a key risk factor for cancer initiation and progression (Jezierska-Drutel, Rosenzweig & Neumann, 2013) resulting from an imbalance between Reactive Oxygen Species (ROS) production and antioxidant defenses, contributes to cellular damage, apoptosis, lipid peroxidation and interferes with the body’s normal metabolic activity, leading to the occurrence and development of diseases (Visconti & Grieco, 2009; Fiaschi & Chiarugi, 2012). Malondialdehyde (MDA) is one of the end products of lipid peroxidation and it is also formed as a product of the cyclooxygenase reaction in prostaglandin metabolism.

Intracellular compartments such as mitochondria, is the major site of ROS production (Poyton et al., 2009). Enzymes involved in ROS-generating chemical reactions are peroxidases, Nicotinamide adenine dinucleotide phosphate oxidase (NOX), xanthine oxidase (XO), lipoxygenases (LOXs), glucose oxidase, myeloperoxidase (MPO), nitric oxide synthase, and cyclooxygenases (COXs) (Kulkarni, Kuppusamy & Parinandi, 2007).

The oxidation of NADPH to NADP^+^ catalyzed by NADPH oxidase generates superoxide radical from oxygen. NADPH oxidase enzyme present in cytoplasmic membrane of phagocytic cells was described first as an enzyme involved in the generation of ROS in the phagocytic cells (Rossi & Zatti, 1964). This enzyme comprises two membrane-bound proteins (p22phox and gp91phox), three cytosolic proteins (p67phox, p47phox, and p40phox), and a small G-protein Rac. Gp91phox and p22phox form a heterodimer that is bound to the plasma membrane. The p22phox subunit is coded by the CYBA (cytochrome b245 alpha) gene, which is mapped to chromosome 16q24.3 (Powell et al., 2002). Genetic factors might regulate NADPH-oxidase-driven O2- production. Several polymorphisms in the NADPH oxidase encoding gene have been described, some of which have been associated with increased (San José et al., 2004) or diminished NOX activity (Guzik et al., 2000), as well as reduced ROS generation (Schirmer et al., 2008; Bedard et al., 2009).

To date little is known about the association of -930 A/G polymorphism (rs9932581) located in the promoter and the 242 C/T polymorphism (rs4673) located in the exon 4 of the CYBA gene, and the level of oxidative stress in BC patients. Therefore, the present study was aimed to examine the importance and association of the functional polymorphisms of CYBA (-930 A/G and 242 C/T) with the oxidative stress in BC development and progression.

## Materials and Methods

### Study population

In our study, a total of 600 subjects were enrolled comprising of 300 histopathologically confirmed female patients with breast cancer and the control group included 300 unrelated healthy women with no self-reported history of any cancer. The study followed the Helsinki declaration and was approved by Institutional Ethics Committee, MNJ Institute of Oncology & Regional Cancer Centre. Patients with breast cancer were enrolled from the Department of Radiation Oncology, MNJ Regional Cancer Centre, Hyderabad from August 2013 to August 2017 and during the same time controls subject were enrolled from the local population and women with any other cancer or other systemic inflammatory disease were excluded from the case and control group.

All subjects were explained about the purpose of the study and were ensured that the information collected from them would be confidential. Subsequently written informed consent to participate in the study was obtained from each individual. Each subject completed a questionnaire on their demographic characteristics, area of living, lifestyle habits such as tobacco use and alcohol consumption. Clinical characteristics such as tumor size, stage of the cancer, axilliary lymph node involvement and metastasis were collected via medical records with the help of medical oncologist.

### Sample collection

Following an overnight fast 4 ml of blood sample was collected by antecubital venipuncture in EDTA vaccutainer from each individual for estimation of MDA & genomic DNA extraction.

### Plasma MDA levels estimation

Lipid peroxidation, as evidenced by the formation of malondialdehyde (MDA), was assayed by the method described previously (Gavino et al., 1981; Rajesh et al., 2011). Briefly, to 0.5 ml of freshly obtained plasma an equal volume of 0.9% saline and trichloroacetic acid (TCA) was added and incubated at 37 °C for 20 min, and centrifuged for 10 min at 3,000 rpm. A total of 0.25 ml of thiobarbituric acid (TBA) was added to 1 ml of protein free supernatant (TCA extract) and the reaction mixture was heated for 60 min at 95 °C till a faint pink color develops. After cooling, the color intensity was measured at 532 nm with eppendorf UV 240-Spectrophotometer. 1,1,3,3-Tetraethoxypropane(1–100 nmol/ml) was used as the standard. The lipid peroxidation activity was expressed in “nano moles” of MDA equivalents/ml of standard 1,1,3,3-Tetraethoxypropane.

### Genomic DNA extraction and genotyping analysis

Genomic DNA was isolated from blood sample using a non-enzymatic method (Miller, Dykes & Polesky, 1988). Polymorphic regions in the CYBA gene were identified by Allele- specific polymerase chain reaction (PCR) and PCR- Restriction fragment length polymorphism (RFLP) assays for -930 A/G and 242 C/T polymorphisms respectively. Cases and controls were randomized during genotyping and 10% of the samples were genotyped in duplicate to assess the genotyping error rate. Concordance of genotypes were 100%.

### Statistical analysis

Demographic, clinical, and biochemical variables are expressed as the mean ± SD. All statistical tests were two-sided, a *P*-value lower than 0.05 was considered statistically significant. For comparison of continuous variables in demographic data between controls and breast cancer patients, Student’s *t*-test was performed. Observed genotype frequencies were tested for deviation from Hardy-Weinberg equilibrium with the chi-square goodness-of-fit test (*χ*^2^). Risk estimates were calculated for co-dominant, dominant and recessive genetic models using SNPStats. Odds ratios (OR) and their 95% confidence intervals (CI) were estimated using a univariate analysis. Linkage Disequilibrium (LD) plots were generated using Haploview (v.4.2) software. Multifactor dimensionality reduction (MDR) analysis was performed to identify high-order interaction models that were associated with BC risk using open-source MDR software (v.2.0 beta 8.4).

### Bioinformatics analysis

Prediction of presumptive changes in transcription factor binding sites (TFBS) caused by nucleotide alterations in the promoter region was performed with AliBaba software 2.1 (http://gene-regulation.com/pub/programs/alibaba2/index.html) (Grabe, 2002). Pre-mRNA secondary structure prediction of 242 C/T polymorphic variants was carried out using the ViennaRNA fold webserver (http://rna.tbi.univie.ac.at/cgi-bin/RNAWebSuite/RNAfold.cgi) online tool (Zuker & Stiegler, 1981). The 3D models for CYBA wild type and variant protein with 242 C/T SNP were generated using homology modeling tool I-TASSER (https://zhanglab.ccmb.med.umich.edu/I-TASSER/) (Roy, Kucukural & Zhang, 2010).

## Results

The baseline characteristics are summarised in Table 1. In the present study, the frequency of individuals with lifestyle habits such as, mixed diet (Non-vegetarian), habit of smoking and alcohol consumption were found to be high with breast cancer (*p* < 0.05).

**Table 1:** Baseline characteristics of controls and breast cancer cases.

Characteristics	**Controls***N* = 300 (%)	**Cases***N* = 300 (%)	**OR(95% CI)**	*p*
Age (years) (mean ± SD)	46.34 ± 7.97	47.98 ± 10.8	–	**0.034**
Lifestyle habits				
Vegetarian Diet	87 (29)	43 (14.34)		
Non-vegetarian Diet	213 (71)	257 (85.56)	2.44 (1.62–3.67)	**<0.005**
Non-smokers	273 (91)	245 (81.66)		
Smoker	27 (9)	55 (18.34)	2.27 (1.38–3.71)	**0.0004**
Non-alcoholics	243 (81)	179 (59.6)		
Alcoholics	57 (19)	121 (40.4)	2.88 (1.99–4.16)	**<0.001**

**Notes.**

OR odds ratio CI Class interval * *p*-value by Student’s *t* test (continuous variables) *χ*^2^ test categorical variables

The genotypic and allele frequency distribution of the CYBA -930 A/G polymorphism is represented in Table 2. In the present study the GG genotype was significantly higher in cases and was found to be associated with an increased risk of BC compared to homozygotic AA genotype carriers (OR 2.15, 95% CI [1.16–3.98], *p* = 0.034). The allelic distribution has revealed that the prevalence of the G-allele was significantly higher in cases and conferred increased risk for breast cancer compared to A-allele (OR 1.27, 95% CI [1.01–1.6], *p* = 0.035).

**Table 2:** Distribution of genotype and allele frequencies of CYBA -930 A/G polymorphism in controls and breast cancer patients.

**Model**	**Genotype**	**Controls***N* (%)	**Cases***N* (%)	**OR (95% CI)**	*χ*^**2**^*p*-value
Co-dominant	A/A	85 (28.3)	62 (20.7)	1.00	0.034^*^
	A/G	192 (64)	202 (67.3)	1.44 (0.98–2.11)	
	G/G	23 (7.7)	36 (12)	**2.15 (1.16–3.98)**	
Dominant	A/A	85 (28.3)	62 (20.7)	1.00	0.029^*^
	A/G−G/G	215 (71.7)	238 (79.3)	**1.52 (1.04–2.21)**	
Recessive	A/A−A/G	277 (92.3)	264 (88)	1.00	0.074
	G/G	23 (7.7)	36 (12)	1.64 (0.95–2.85)	
Over dominant	A/A−G/G	108 (36)	98 (32.7)	1.00	0.39
	A/G	192 (64)	202 (67.3)	1.16 (0.83–1.62)	
Log-additive	–	–		1.46 (1.09–1.94)	0.0094
Allele	A	362 (0.6)	326 (0.54)	1.00	0.035^*^
	G	238 (0.4)	274 (0.46)	**1.27 (1.01–1.6)**	
HWE(p)		**<0.0001**	**<0.0001**		

**Notes.**

* *χ*^2^*p*-value <0.05 is considered statistically significant.

The genotype and allele frequency distribution of CYBA 242 C/T polymorphism among the controls and patients with breast cancer is presented in Table 3. Under the dominant model, carriers of at least one minor allele T (CT + TT) were found to be associated with a significantly increased risk of BC compared to major allele homozygotic (CC) carriers (OR 1.42, 95% CI [1.02–1.98], *p* = 0.036). The allelic association revealed that the minor allele T of 242 C/T polymorphism was associated with an increased risk of BC (OR 1.36, 95% CI [1.04–1.78], *p* = 0.02).

**Table 3:** Distribution of genotype and allele frequencies of CYBA 242 C/T polymorphism in controls and breast cancer patients.

**Model**	**Genotype**	**Controls***N* (%)	**Cases***N* (%)	**OR (95% CI)**	*χ*^**2**^*p*-value
Co-dominant	C/C	197 (65.7)	172 (57.3)	1.00	0.1
	C/T	82 (27.3)	99 (33)	1.38 (0.97–1.98)	
	T/T	21 (7)	29 (9.7)	1.58 (0.87–2.88)	
Dominant	C/C	197 (65.7)	172 (57.3)	1.00	**0.036**
	C/T−T/T	103 (34.3)	128 (42.7)	**1.42 (1.02–1.98)**	
Recessive	C/C−C/T	279 (93)	271 (90.3)	1.00	0.24
	T/T	21 (7)	29 (9.7)	1.42 (0.79–2.55)	
Over dominant	C/C−T/T	218 (72.7)	201 (67)	1.00	0.13
	C/T	82 (27.3)	99 (33)	1.31 (0.92–1.86)	
Log-additive	–	–	–	**1.31 (1.02–1.68)**	**0.036**
Allele	C	476 (0.79)	443 (0.74)	1.00	**0.02**
	T	124 (0.21)	157 (0.26)	**1.36 (1.04–1.78)**		**0.02**
HWE(p)		0.16	**0.027**		

**Notes.**

*χ*^2^*p*-value <0.05 is considered statistically significant.

We further have analysed the haplotype frequencies with respect to CYBA gene polymorphisms in association with risk of breast cancer. Our analysis has revealed a total of four haplotypes as shown in Table 4. Comparison of haplotype frequencies between controls and BC patients revealed a significant difference in haplotype frequencies, where -930G/242C and -930G/242T combinations were found to be significantly associated with an increased risk of breast cancer by more than 1.44 fold (95% CI [1.00–2.07]; *p* = 0.05) and 1.56 (95% CI [1.11–2.20]; *p* < 0.05) respectively compared with the common haplotype (-930A/242C).

**Table 4:** Haplotype frequencies of CYBA -930 A/G and 242 C/T polymorphisms between Controls and BC patients.

**Haplotype**^a^	**Overall(*N* = 600)**	**Controls(*N* = 300)**		**Cases(*N* = 300)**	**OR (95% CI)**	*p*-value
A-C	0.5116	0.547	0.4752	1.00	–
**G-C**	**0.2542**	**0.2463**	**0.2632**	**1.44 (1.00–2.07)**	**0.05**
**G-T**	**0.1724**	**0.1503**	**0.1503**	**1.56 (1.11–2.20)**	**0.011**
A-T	0.0617	0.0563	0.0682	1.40 (0.75–2.59)	0.29

**Notes.**

a Order of SNPs in CYBA gene haplotypes: -930 A/G, 242 C/T; OR, Odds ratio; CI, Class interval.

* Interactive Chi-Square *p*-value < 0.05 is statistically significant.

**Figure 1:**
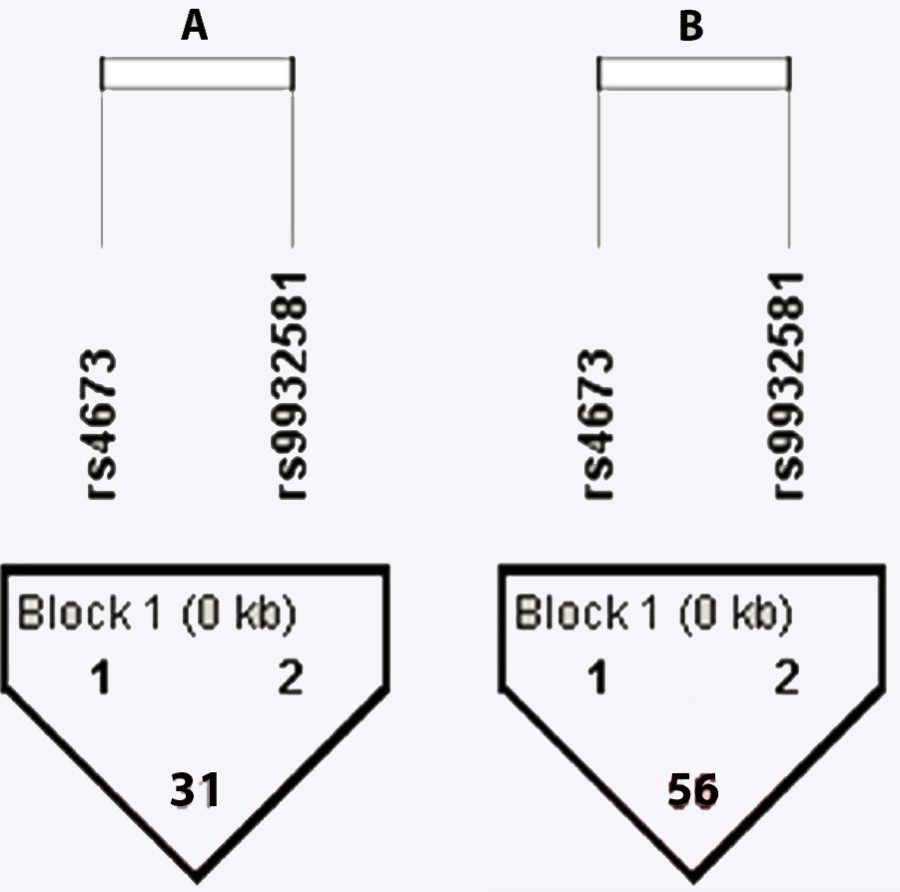
Plot of pair-wise linkage disequilibrium (LD) analysis of SNPs of CYBA genes in controls and BC patients. (A) LD plot of controls (B) LD plot of Cases. *D*′ values are shown in the plot. A value of 100 represents maximum possible linkage disequilibrium.

**Figure 2:**
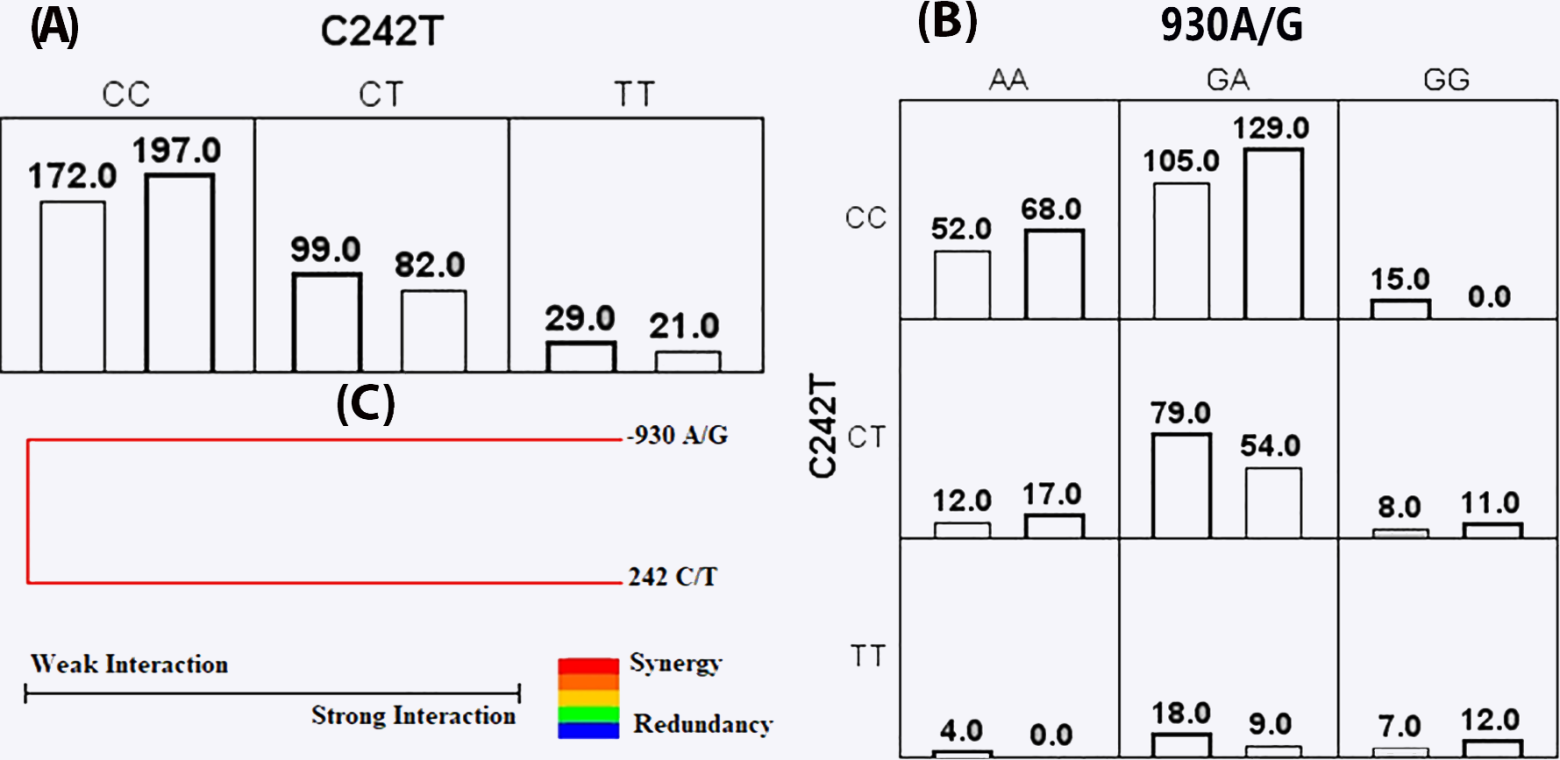
Multifactor dimensionality reduction (MDR) analysis of CYBA gene polymorphisms in association with breast cancer. (A) Univariate and (B) Bivariate analysis—In each block, the first bar represents the breast cancer patients group and the adjacent bar represents the control group respectively. (C) Interaction dendrogram—The interaction dendrogram was used to confirm, visualize, and interpret the interaction model. The colours used to depict the degree of synergy, ranging from red (highest information gain) to blue (highest information redundancy).

Pairwise LD was computed for CYBA -930 A/G and 242 C/T polymorphism in cases and controls separately. LD plots revealed a moderate LD (*D*′ = 56) between the markers in BC patients and a weak LD (*D*′ = 31) between the markers in controls as shown in the Fig. 1. Further, MDR analysis with respect to CYBA gene polymorphism has shown that 242C/T polymorphism was the best single locus model with a significant risk for breast cancer. The bivariate model showed a strong interaction between -930 A/G and 242 C/T polymorphisms as seen in Fig. 2. Our analysis has revealed a synergistic interaction between SNPs (gain of information).

**Figure 3:**
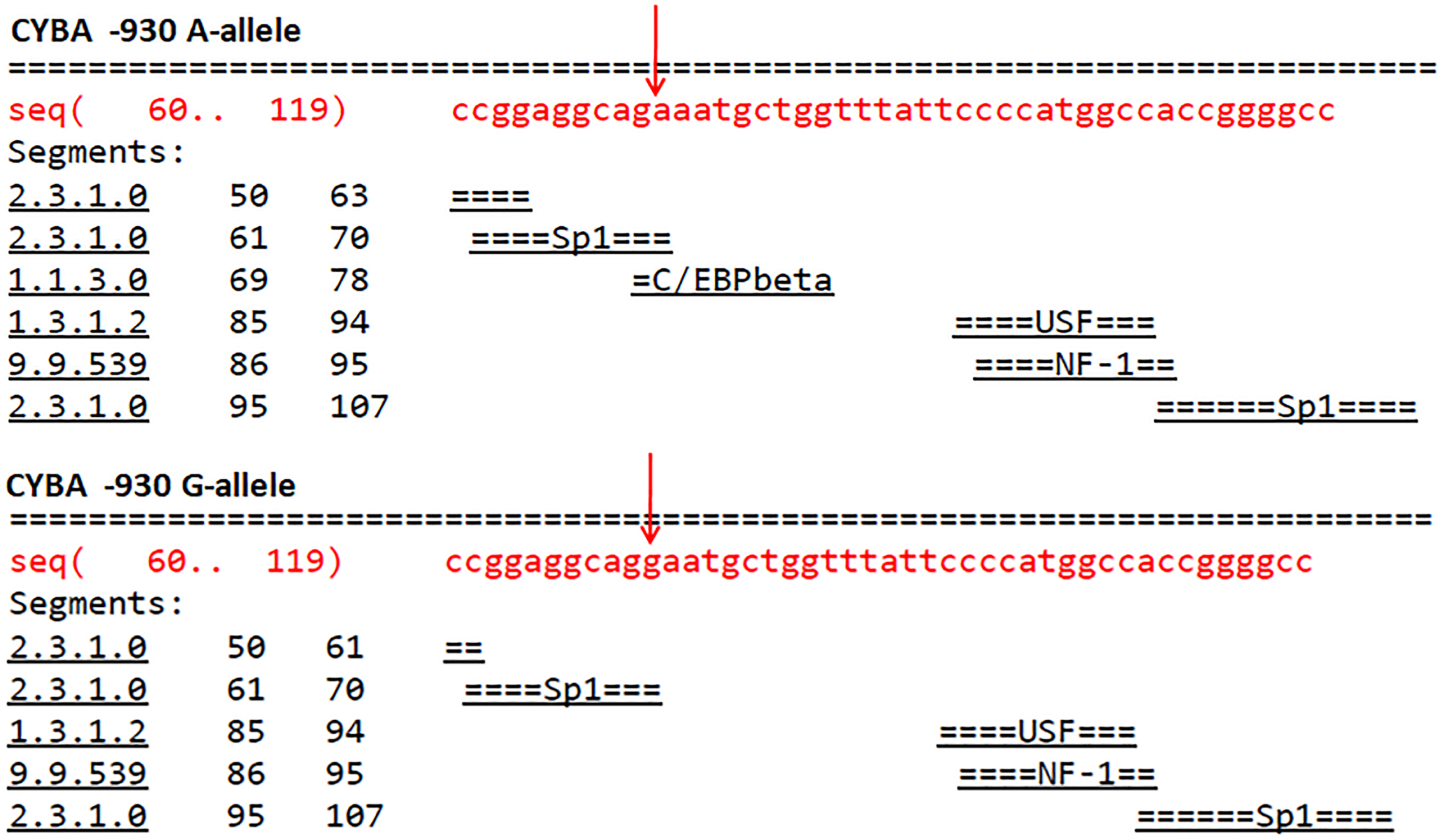
Effect of the CYBA -930 A/G polymorphism on transcription factor binding sites.

**Figure 4:**
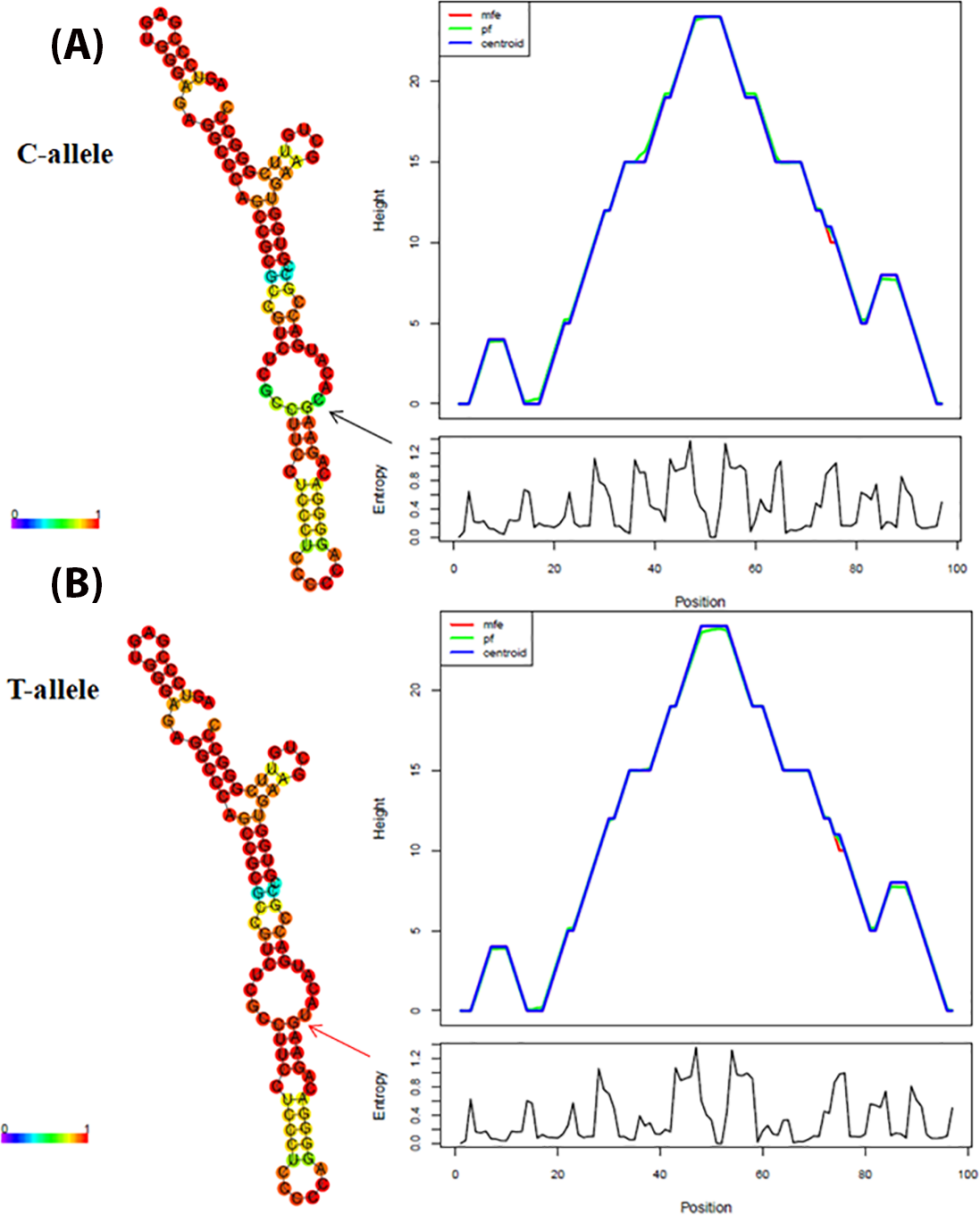
Computational analysis of CYBA 242 C/T polymorphism based pre-mRNA secondary structures. Predicted minimal free energy based RNA structure of (A) major (C-allele) and (B) minor (T-allele) alleles of 242 C/T polymorphism using the RNA fold program in the Vienna RNA package (Zuker algorithm). Structure colours encode base-pair probabilities and arrow denotes the location of polymorphism. The mountain plot is a *XY*-graph that represents a secondary structure including MFE structure, the thermodynamic ensemble of RNA structures (pf), and the centroid structure in a plot of height versus position. “mfe” represents minimum free energy structure; “pf” indicates partition function; “centroid” represents the centroid structure.

**Figure 5:**
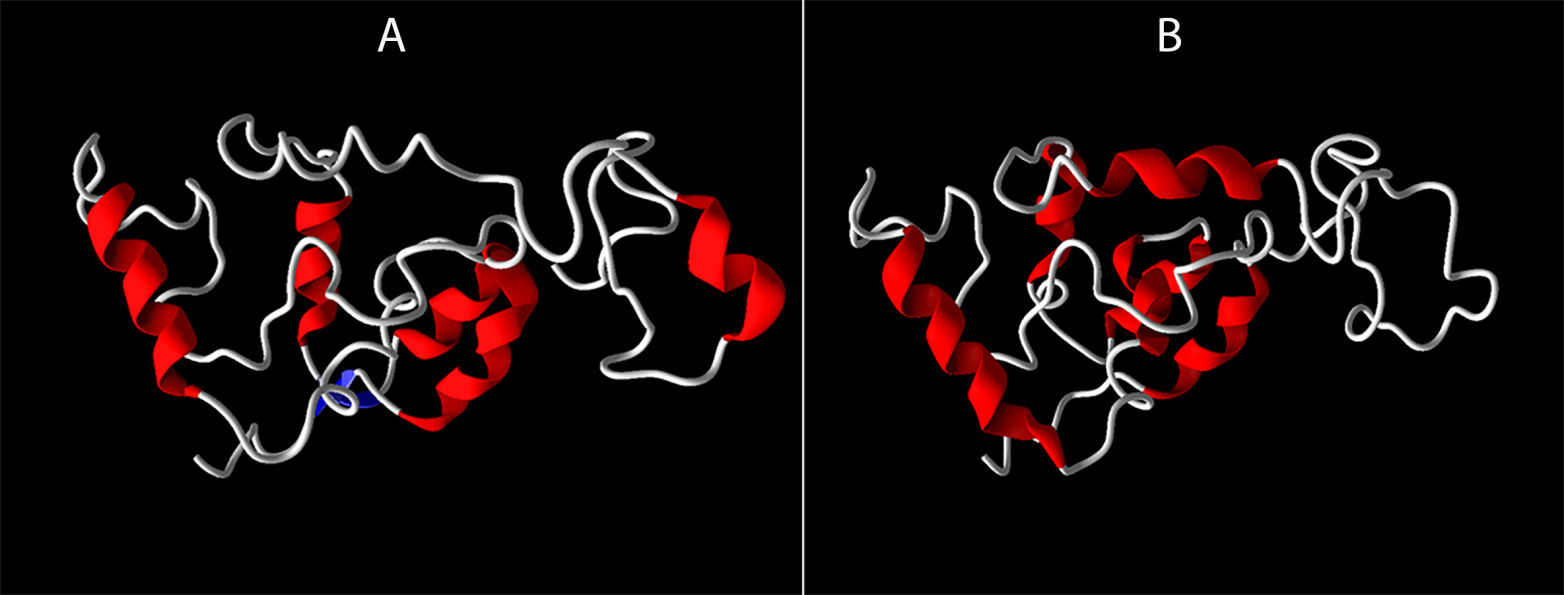
3D structures of CYBA 242 C/T polymorphic variants as predicted by I-TASSER. The 3D structures of the CYBA 242 C/T polymorphic variants were modeled on I-TASSER server. (A) displays the wildtype structures, and (B) exhibits the relevant variant structure.

Furthermore, the TFBS analysis with respect to -930 A/G promoter polymorphism has revealed that substitution of A nucleotide by G leads to a loss of C/EBPbeta site as depicted in Fig. 3. The comparison of the wild type and variant pre-mRNA secondary structures with respect to 242 C/T polymorphism is given in Fig. 4, wherein, the stability, as depicted by minimum free energy (MFE) change has revealed that the T-allelic structure had an MFE of −37.61 Kcal/mol and the C-allelic structure had an MFE of −37.91 Kcal/mol respectively. In addition, an altered 3D structure was also observed corresponding to loss of cavities with respect to variant structure when compared to wild type structure as seen in Fig. 5 (Table 5).

**Figure 6:**
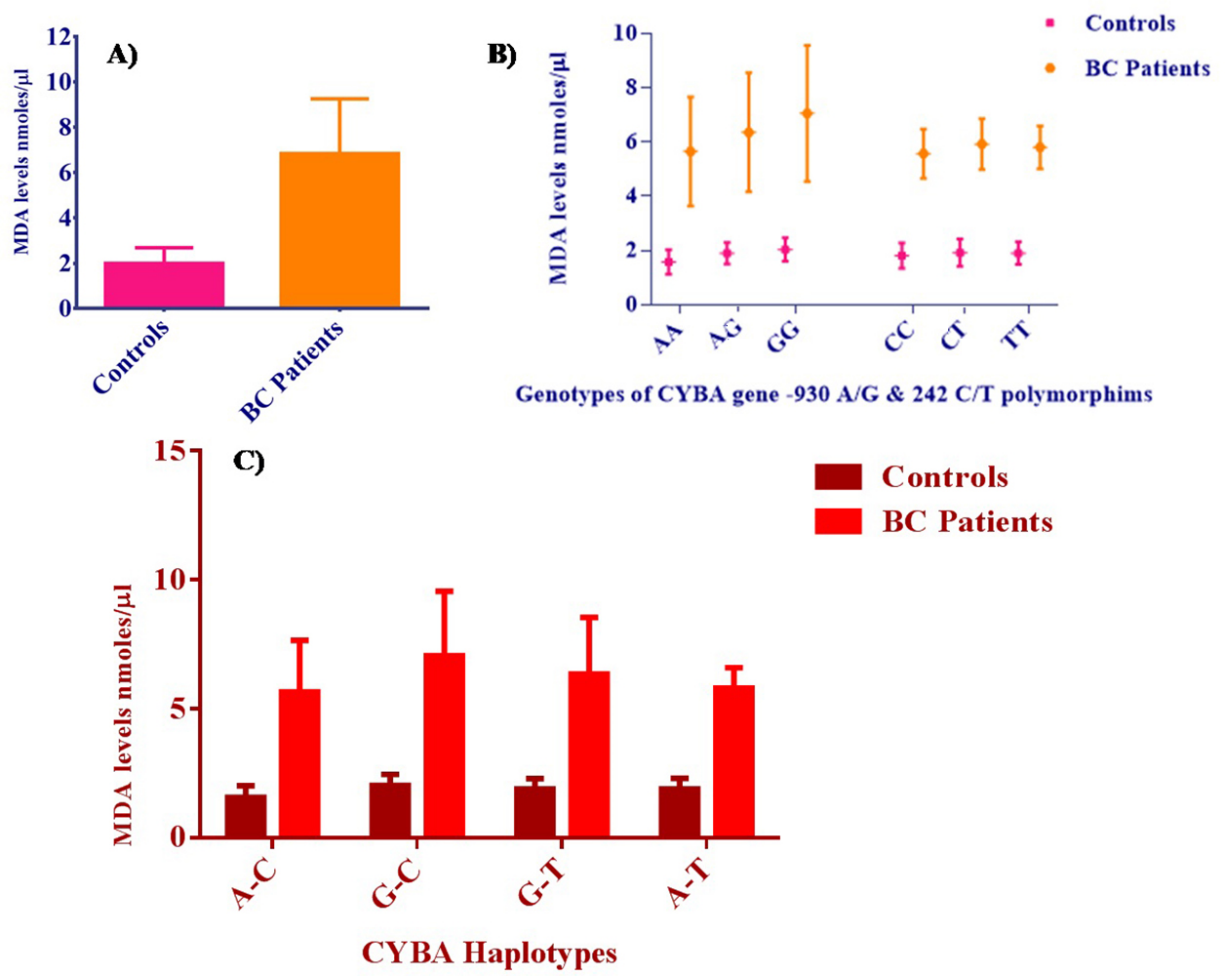
MDA levels in controls and breast cancer patients. (A) Malondialdehyde (MDA) levels in the control group and breast cancer patients (B) MDA levels with respect to CYBA polymorphic genotypes and (C) MDA levels with respect to CYBA gene haplotypes in controls and breast cancer patients.

The plasma MDA levels were measured in all the subjects in the present study, our results revealed that patients with breast cancer had significantly higher MDA levels (6.84 ± 2.42 nmoles/µl) compared to the control (2 ± 0.69 nmoles/µl) group as depicted in Fig. 6A. Further, MDA levels were stratified with respect to CYBA genotypes, where we found that individuals with GG genotype of -930 A/G polymorphism had higher MDA levels compared to those with AA genotype as shown in Fig. 6B. Furthermore, the MDA levels with respect to CYBA gene haplotypes has shown that -930G/242C haplotype combination was associated with higher MDA levels in breast cancer patients compared to other haplotypes at *p* < 0.05 as summarized in Fig. 6C.

## Discussion

Breast cancer is a common disease worldwide and also one of the leading cause of cancer deaths in India (Ferlay et al., 2015; Malvia et al., 2017). Breast carcinogenesis involves a cascade of multiple intracellular mechanisms such as genetic alterations, signal transduction pathways, etc, (Kurose et al., 2001). However, it also depends on the oxidative stress (OS) and the predominance of endogenous antioxidant system for manifestation of disease. Oxidative stress induces uncontrolled lipid peroxidation with produces aldehyde end-products, such as free fatty acids, malondialdehyde (MDA) that might cause cell injury and death. In addition, cancer initiation and progression have also been shown to be associated with oxidative stress by causing DNA mutations or inducing DNA damage, genome instability, and cell proliferation (Srivastava et al., 2009; Visconti & Grieco, 2009; Wang et al., 2011; Wu et al., 2017).

In the present study, a higher frequency of breast cancer patients with smoking or alcohol habits was observed. Multiple reports have also shown that habit of smoking and alcohol consumption were associated with increased risk for breast cancer as they are more exposed to free radicals leading to oxidative damage to lipids, proteins and DNA that may aid in cancer progression (Kumari et al., 2018; Scheideler & Klein, 2018). In contrast, several reports have been inconsistent, wherein no significant association was observed with respect to smoking and alcohol consumption in breast cancer patients (Byrne, Rockett & Holmes, 2002; Allen et al., 2009; Gathani et al., 2017).

**Table 5:** Cavity differences between the structures of CYBA 242C/T polymorphic variants.

**Cavity**	Volume (A^∘^3)	
	**Wild type**	**Variant type**
1	53.248	132.09
2	51.2	28.67
3	22.01	17.92
4	19.96	13.312
5	18.94	–

Alteration in expression of enzyme system that produces ROS such as NADPH oxidase (NOX) has been shown to be an important susceptibility factor for cancer (Arcucci et al., 2016). The most significant sources of ROS are NOXs, which include two membrane-bound subunits Nox2 and p22phox. The p22phox encoded by the CYBA gene has several functional polymorphisms. In view of the above, in this study we attempted to determine the association of oxidative stress with -930 A/G and 242 C/T polymorphisms of CYBA gene that encodes p22phox subunit of NADPH oxidase among controls and patients with breast cancer to understand its role in the development and progression of breast cancer. The -930 A/G functional SNP located at the promoter region in a dual-luciferase reported assay system has revealed that the G allele was found to be associated with a 30% increase in promoter activity. Furthermore, the frequency of the G allele was higher than the A allele in hypertensive individuals (Moreno et al., 2003). Recent large population study on -930 A/G polymorphism has also reported that the GG genotype confers susceptibility for hypertension (Kokubo et al., 2005). Therefore, we have investigated the association between this SNP in association with breast cancer risk. In the present population the G-allele was found to be significantly higher in breast cancer patients compared to healthy controls conferring a 1.27-fold risk towards breast cancer. The promoter region SNPs affects gene expression by altering promoter activity, transcription-factor binding, DNA methylation and histone modifications (Deng et al., 2017). Interactions between transcription factors (TFs) and target binding sites determine the expression of genes. Since the -930 A/G polymorphism has a potential binding site for C/EBP (CCAAT/enhancer-binding protein) transcription factors it has been speculated that it might modulate CYBA transcriptional activity (San José et al., 2004). Our insilco analysis on transcription-factor binding sites with respect to -930 A/G polymorphic variants revealed that the substitution of A by G results in the loss of repressor C/EBPbeta transcription factor site that might increase transcriptional activity.

The C242T polymorphism has been demonstrated to be related to multiple diseases (Guzik et al., 2000; San José et al., 2008; Vibhuti et al., 2010; Schreiber et al., 2011; Zhou & Zhao, 2015). Results of the present study had showed that individuals with the CT/TT genotype of 242 C/T polymorphism had a 1.42-fold higher risk for breast cancer compared to those with the CC genotype. Our finding was consistent with reports showing significant association with vascular disease (Ito et al., 2000). The C242T polymorphism located in exon 4 encodes a CAC →TAC codon change thus resulting in a non-conservative substitution of His72 for a tyrosine residue that may alter the haem-binding site of the p22phox protein (Tahara et al., 2008; Fu et al., 2016). Finding 3D structure of proteins is helpful in predicting the impact of SNPs on the structural level and in showing the degrees of alteration. Our analysis has shown an altered 3D structure with a change of histidine residue in the variant protein that might contribute to functional impairment.

MDA is a naturally occurring endogenous product of lipid peroxidation and prostaglandin biosynthesis, but is mutagenic and carcinogenic. Oxidative stress as measured by an increase in MDA levels was established in gastric, colorectal adenomas, prostate and oral cancer (Bakan et al., 2002; Leuratti et al., 2002; Zhang et al., 2008; Chole et al., 2010). In this study we have also demonstrated an increase in lipid peroxidation due to oxidative stress in breast cancer patients. Previous studies have also reported increased levels of MDA in breast cancer patients compared to healthy controls (Gönenç et al., 2001; Gönenç et al., 2006; Yeh et al., 2005) suggesting that elevated oxidative stress contributes to increased risk for breast cancer development and progression. Further, comparison of MDA levels with respect to CYBA gene haplotypes revealed that -930G/242C and -930G/242T haplotype carriers in the patients with breast cancer showed higher MDA levels than other haplotypes; this could be in line with observation that states presence of G-allele could increase the transcriptional activity, elevating ROS production resulting in oxidative stress in breast cancer patients.

There are several limitations in this study. The foremost limitation to our study concerns the use of limited sample size, which prevented us from drawing causal relationships. Owing to its importance as an oxidative stress indicator, we have measured MDA levels; however, MDA levels alone are not a sole indicator of oxidative stress and we have not directly quantified the NADPH oxidase activity. Further studies on CYBA gene polymorphisms/haplotypes, along with different oxidative stress markers, should be done in a multicenter, multi-ethnic population and with a large number of patients to strengthen our findings.

## Conclusion

In conclusion, our results suggest that the individuals with GG genotype of -930 A/G polymorphism, -930G/242C and/or -930G/242T haplotypes of CYBA gene may predispose to increased oxidative stress. Therefore, more attention should be paid to oxidative stress-related pathological manifestations in individuals with the risk genotype/haplotype, as it plays an important role in development and progression of breast cancer.

##  Supplemental Information

10.7717/peerj.5509/supp-1 Supplemental Information 1 Raw data

## Additional Information and Declarations

### Data Availability

The following information was supplied regarding data availability: The raw data are provided in a Supplemental File.
